# Characterization of the complete chloroplast genome of sorrel (*Rumex acetosa*)

**DOI:** 10.1080/23802359.2018.1501297

**Published:** 2018-08-17

**Authors:** Lingjian Gui, Shaofeng Jiang, Huaiping Wang, Dongxin Nong, Yingying Liu

**Affiliations:** a Guangxi Botanical Garden of Medicinal Plants, Nanning, China;; b Guangdong Province Key Laboratory of Microbial Signals and Disease Control, Department of Plant Pathology, South China Agricultural University, Guangzhou, China;; c Cardiovascular disease hospital affiliated to Qingdao University, Shandong, China

**Keywords:** *Rumex acetosa*, Chloroplast genome, Illumina sequencing, Phylogenetic analysis

## Abstract

*Rumex acetosa*, known as sheep’s sorrel, red sorrel, sour weed, and field sorrel, is a species of flowering plant in the buckwheat family Polygonaceae. In this study, the complete chloroplast (cp) genome of *R. acetosa* (Rumiceae) was determined through Illumina sequencing method. The complete chloroplast genome of *R. acetosa* was 160,269 bp in length and contained a pair of IR regions (30,503 bp) separated by a small single copy region (13,128 bp) and a large single copy region (86,135 bp). This cp genome is encoded with 129 genes including 83 protein-coding genes, 36 tRNA genes, and 8 ribosomal RNA genes. The overall GC content of *R. acetosa* cp genome is 37.2%. By phylogenetic analysis using Bayesian method, *R. acetosa* showed the closest relationship with other 2 Rumiceae species, *Rheum palmatum* and *Oxyria sinensis*.

*Rumex acetosa* L. (Polygonaceae), a dioecious plant which has a multiple sex chromosome system (Shibata et al. 1999, 2000), is a perennial herb, commonly known as sheep’s sorrel, red sorrel, sour weed, and field sorrel (Lee et al. 2005). As a traditional medicine plant, it was shown to have some pharmacological activities, including anti-inflammatory, antioxidant (Wegiera et al. 2007), anti-tumor, antibacterial, antiviral, and anti-fungal properties (Taylor et al. 1996; Demirezer et al. 2001; Lee et al. 2005). Meanwhile, its leaves are widely used in sauces and salads (Ahmad et al. 2006). With an aim to retrieve valuable cp molecular markers, indels, and SSRs by comparative analyses with other Rumiceae cp genomes, we assembled and analyzed the chloroplast genome of *R. acetosa* based on the next-generation sequencing method.

Leaves from *R. acetosa* were collected in Sierra Nevada, Granada, Spain (37°10′N, 3°17′W). Both extracted DNA and this species voucher were stored at Guangxi Botanical Garden of Medicinal Plants. Sequencing was done on the Illumina Hiseq-2500 platform to produce 150 bp paired-end reads (BGI Tech, Shenzhen, China). After reads quality filtration, the clean reads were assembled by SPAdes 3.6.1 (Bankevich et al. 2012). We used chloroplast genome of *Rheum palmatum* (accession number: KR816224) (Fan et al. 2015) as a reference sequence to align the contigs and identify gaps. To fill the gap, Price (Ruby et al. 2013) and MITObim v1.8 (Hahn et al. 2013) were applied, and Bandage (Wick et al. 2015) was used to identify the borders of the IR, LSC, and SSC regions. The complete sequence was primarily annotated by Geseq (Tillich et al. 2017) and Plann (Huang et al. 2015) combined with manual correction. All tRNAs were confirmed using the tRNAscan-SE search server (Lowe et al. 1997). Protein-coding genes were verified by BLAST search on the NCBI website (http://blast.ncbi.nlm.nih.gov/) and manual correction for start and stop codons was conducted. The circular cp genome map was drawn using OrganellarGenomeDRAW (Lohse et al. 2007). This complete chloroplast genome sequence together with gene annotations were submitted to GenBank under the accession number of MH359405.

The chloroplast genome of *R. acetosa* is a typical quadripartite structure with a length of 160,269 bp. The whole cp genome contains a large single-copy (LSC) region of 86,135 bp, a small single-copy (SSC) region of 13,128 bp, and 2 inverted repeat (IRs) regions of 30,503 bp. The cp genome possesses 129 genes, including 83 protein-coding genes (78 PCG species), 8 ribosomal RNA genes (4 rRNA species) and 36 tRNA genes (30 tRNA species). The overall GC content of the cp genome is 37.2%. The genome structure, gene order, and GC content are similar to other Rumiceae cp genomes.

For phylogenetic analysis assessing the relationship of this plastid, we selected other 43 Caryophyllales cp genomes including Caryophyllaceae (15 taxa), Amaranthaceae (2 taxa), Chenopodiaceae (11 taxa), Aizoaceae (2 taxa), Cactineae (5 taxa), Polygonaceae (4 taxa), and Droseraceae (4 taxa) to construct a genome-wide alignment. We considered plastids of the Fagales as the outgroup. The genome-wide alignment of all cp genomes was done by HomBlocks (Bi et al. 2018), resulting in a total of 45,627 positions. The whole genome alignment was analyzed by PhyloBayes ver. 3.3 (Lartillot et al. 2009) under the CAT-GTR + Γ model that accounts for across-site heterogeneities. Four independent MCMC analyses were run for 1,000,000 cycles in PhyloBayes. Convergence was verified based on time-series plots of the likelihood scores using Tracer (http://tree.bio.ed.ac.uk/software/tracer/). The first 25% cycles were discarded as burn-in, and the maximum clade credibility (MCC) tree was constructed in TreeAnnotator v1.8.0 (Rambaut et al. 2012) depicting the maximum sum of Bayesian posterior probabilities (BPPs). The resulting tree was represented and edited using FigTree v1.4.1 (http://tree.bio.ed.ac.uk/software/fgtree/). As shown in Figure 1, the phylogenetic positions of these 44 cp genomes were successfully resolved with full BPPs supports except for 6 nodes. *Rumex acetosa* belongs to the Polygonaceae as expected and exhibited the closest relationship with other 2 Rumiceae species, *Rheum palmatum* and *Oxyria sinensis*.

**Figure 1.**
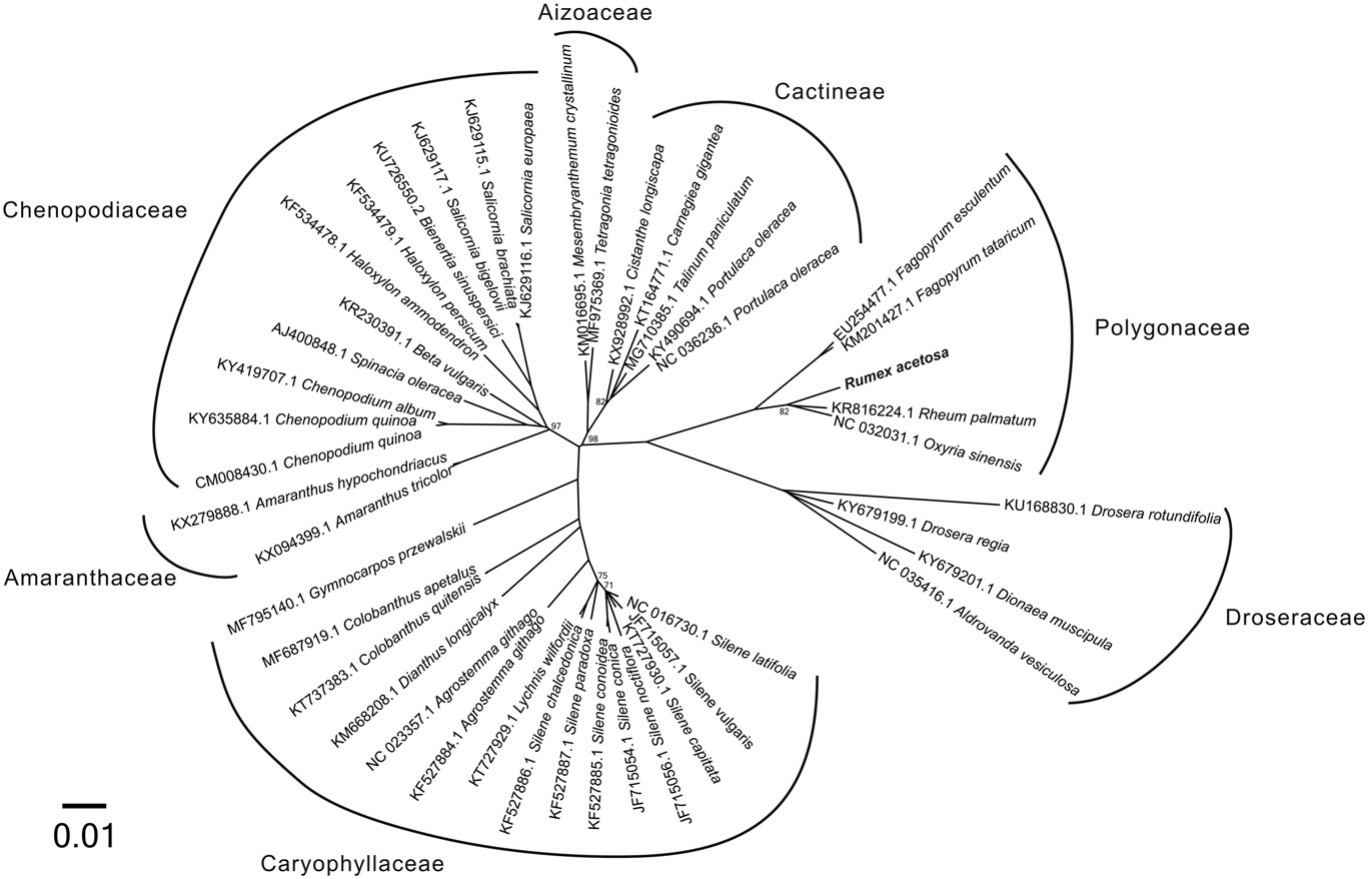
Phylogenetic tree yielded by Bayesian analysis of 44 *Caryophyllales* cp genomes. Bayesian consensus tree is shown with support indicated by numbers at branches, representing the percentage of Bayesian posterior probabilities (BPPs). Fully phylogenetical resolved nodes are not labeled.

## References

[CIT0001] Ahmad S, Ullah F, Sadiq A, Ayaz M, Imran M, Ali I, Zeb A, Ullah F, Shah MR 2016 Chemical composition, antioxidant and anticholinesterase potentials of essential oil of Rumex hastatus D. Don collected from the North West of Pakistan. BMC Complementary Altern Med. 16(1):29. 10.1186/s12906-016-0998-z PMC4727414 26810212

[CIT0002] Bankevich A, Nurk S, Antipov D, Gurevich AA, Dvorkin M, Kulikov AS, Lesin VM, Nikolenko SI, Pham S, Prjibelski AD, et al. 2012 SPAdes: a new genome assembly algorithm and its applications to single-cell sequencing. JComp Bio. 19:455–477. 10.1089/cmb.2012.0021 PMC3342519 22506599

[CIT0003] Bi G, Mao Y, Xing Q, Cao M 2018 HomBlocks: a multiple-alignment construction pipeline for organelle phylogenomics based on locally collinear block searching. Genomics. 110:18–22. 28780378 10.1016/j.ygeno.2017.08.001

[CIT0004] Demirezer LÖ 2001 The structures of antioxidant and cytotoxic agents from natural source: anthraquinones and tannins from roots of Rumex patientia. Phytochemistry. 58:1213–1217. 11738410 10.1016/s0031-9422(01)00337-5

[CIT0005] Fan K, Sun X-J, Huang M, Wang X-M 2015 The complete chloroplast genome sequence of the medicinal plant Rheum palmatum L.(Polygonaceae). Mitochondrial DNA Part A. 27:1–2936. 10.3109/19401736.2015.1060448 26153751

[CIT0006] Hahn C, Bachmann L, Chevreux B 2013 Reconstructing mitochondrial genomes directly from genomic next-generation sequencing reads-a baiting and iterative mapping approach. Nucleic Acids Res. 10.1093/nar/gkt371 PMC3711436 23661685

[CIT0007] Huang DI, Cronk QCB 2015 Plann: a command-line application for annotating plastome sequences. Appl Plant Sci. 3:1500026. 10.3732/apps.1500026 PMC4542940 26312193

[CIT0008] Lartillot N, Lepage T, Blanquart S 2009 PhyloBayes 3: a Bayesian software package for phylogenetic reconstruction and molecular dating. Bioinformatics. 25:2286–2288. 19535536 10.1093/bioinformatics/btp368

[CIT0009] Lohse M, Drechsel O, Bock R 2007 OrganellarGenomeDRAW (OGDRAW): a tool for the easy generation of high-quality custom graphical maps of plastid and mitochondrial genomes. Curr Genet. 41(13):e129–e129. 10.1007/s00294-007-0161-y 17957369

[CIT0010] Lee N-J, Choi J-H, Koo B-S, Ryu S-Y, Han Y-H, Lee S-I, Lee D-U 2005 Antimutagenicity and cytotoxicity of the constituents from the aerial parts of Rumex acetosa. Biol Pharm Bull. 28:2158–2161. 16272711 10.1248/bpb.28.2158

[CIT0011] Lowe TM, Eddy SR 1997 tRNAscan-SE: a program for improved detection of transfer RNA genes in genomic sequence. Nucleic Acids Res. 25:955–964. 9023104 10.1093/nar/25.5.955 PMC146525

[CIT0012] Rambaut A, Drummond AJ. TreeAnnotator version 1.6. 1. University of Edinburgh, Edinburgh, UK. Available at: http://beast. bio. ed. ac. uk (2010).

[CIT0013] Ruby JG, Bellare P, DeRisi JL 2013 PRICE: software for the targeted assembly of components of (Meta) genomic sequence data. G3: Genes| Genomes| Genetics. 3:865–880. 23550143 10.1534/g3.113.005967 PMC3656733

[CIT0014] Shibata F, Hizume M, Kuroki Y 1999 Chromosome painting of Y chromosomes and isolation of a Y chromosome-specific repetitive sequence in the dioecious plant *Rumex acetosa*. Chromosoma. 108:266–270. 10460415 10.1007/s004120050377

[CIT0015] Shibata F, Hizume M, Kuroki Y 2000 Differentiation and the polymorphic nature of the Y chromosomes revealed by repetitive sequences in the dioecious plant, *Rumex acetosa*. Chromosome Res. 8:229–236. 10841050 10.1023/a:1009252913344

[CIT0016] Taylor RSL 1996 Antiviral activities of medicinal plants of southern Nepal. J Ethnopharmacol. 53:97–110. 8844464 10.1016/0378-8741(96)01430-4

[CIT0017] Tillich M, Lehwark P, Pellizzer T, Ulbricht-Jones ES, Fischer A, Bock R, Greiner S 2017 GeSeq - versatile and accurate annotation of organelle genomes Nucleic Acids Res. 31(20):3350–3352. 10.1093/nar/gkx391 PMC5570176 28486635

[CIT0018] Wegiera M, Smolarz HD, Wianowska D, Dawidowicz AL 2007 Anthracene derivatives in some species of Rumex L. genus. Acta Soc Bot Pol. 76:103.

[CIT0019] Wick RR, et al. 2015 Bandage: interactive visualization of de novo genome assemblies. Bioinformatics. 10.1093/bioinformatics/btv383 PMC4595904 26099265

